# The principal axes systems for the elastic properties of monoclinic gallia

**DOI:** 10.1038/s41598-020-73970-9

**Published:** 2020-11-10

**Authors:** Marius Grundmann

**Affiliations:** Felix-Bloch-Institut für Festkörperphysik, Fakultät für Physik und Geowissenschaften, Universität Leipzig, Linnéstr. 5, 04103 Leipzig, Germany

**Keywords:** Condensed-matter physics, Materials for devices, Condensed-matter physics

## Abstract

We discuss the principal axes systems of monoclinic and triclinic crystals regarding their elastic properties. Explicit formulas are presented for the orientation of these coordinate systems for monoclinic crystals. In this context, theoretical results from literature on the elastic properties of monoclinic (space group C2/m) gallia and alumina are critically discussed.

## Introduction

The two crystal classes of lowest symmetry are monoclinic and triclinic. In the first, one angle of the unit cell is non-orthogonal, for the latter all angles. Accordingly, the stress-strain relation is fairly complicated and contains 13 or 21 elastic constants, respectively. As already stated by Voigt^1^, there are two principal axis systems for these crystals with regard to their elastic properties. The one system, the ’principal axes of elastic deformation’ (PA-D) is a Cartesian system oriented in a way that a rectangular box cut parallel to the axes reacts to equal normal forces, i.e. hydrostatic pressure, with (generally different) dilations but does not change its (right) angles. These axes are also termed the principal axes of the compression ellipsoid.

The other symmetry adapted system, the ’principal axes of elastic resistance’ (PA-R) is oriented in a way that the same dilation in all directions, preserving the right angles of the box, is evoked by (generally different) normal forces (and zero shear forces).

Recently, monoclinic semiconductors and their strained heterostructures have found high interest in the space group C2/m ${\text {(Al,Ga)}}_2 {\text {O}}_3$ system^2–4^. These materials are promising for device applications, e.g. in high power electronics^5^ and ultraviolet photodetectors^6^. For the calculation of strained heterostructures^7–9^, of course the elastic constants are important input parameters. Various density functional theory based calculations of the elastic constants have been reported for the binary end components, $\beta$-${\text {Ga}}_2 {\text {O}}_3$^10–14,18,24^ and $\theta$-${\text {Al}}_2 {\text {O}}_3$^15–17^. Also, for $\beta$-${\text {Ga}}_2 {\text {O}}_3$ two sets of elastic constants have been determined experimentally^18,23^. We find it helpful to derive here analytical formulas for the orientation of the PA-D and PA-R coordinate systems. These allow the comparison of elastic symmetry of different materials independent of their absolute compliance/stiffness. The different theoretical calculations for the same materials will be critically compared.

## Definition of the crystal system

The crystal is described with respect to a Cartesian coordinate system $\varvec{{\tilde{x}}}=(1,0,0)^{\mathrm {T}}$, $\varvec{{\tilde{y}}}$ and $\varvec{{\tilde{z}}}$. It must be the same as used for the crystal stress-strain relation (12) given below. A vector in this system is denoted as $\varvec{{\tilde{r}}}$.

The lattice vectors of the unit cell are $\varvec{a_1}=(a_{11}, a_{12}, a_{13})^{\mathrm {T}}$, $\varvec{a_2}$ and $\varvec{a_3}$. A vector in the crystal ${\varvec{r}}$ is related to $\varvec{{\tilde{r}}}$ via

1 $$\begin{aligned} {\varvec{r}}= {\varvec{T}} \, \varvec{{\tilde{r}}} \end{aligned}$$

with

2 $$\begin{aligned} {\varvec{T}}= \left( \begin{array}{ccc} a_{11} &{}\quad a_{21} &{}\quad a_{31} \\ a_{12} &{}\quad a_{22} &{}\quad a_{32} \\ a_{13} &{}\quad a_{23} &{}\quad a_{33} \\ \end{array} \right) , \end{aligned}$$

with $$\varvec{a_1}={\varvec{T}} \, \varvec{{\tilde{x}}}$$, $$\varvec{a_2}={\varvec{T}} \, \varvec{{\tilde{y}}}$$, and $$\varvec{a_3}={\varvec{T}} \, \varvec{{\tilde{z}}}$$.

A minimum of six non-zero components is required for the most general case. The standard choice for a triclinic crystal is^19,20^,

3 $$\begin{aligned} \varvec{T_{\mathrm {t}}}= \left( \begin{array}{ccc} a &{}\quad b \, \cos \gamma &{}\quad c_x \\ 0 &{}\quad b \, \sin \gamma &{}\quad c_y \\ 0 &{}\quad 0 &{}\quad c_z \\ \end{array} \right) . \end{aligned}$$

with

4 $$\begin{aligned} c_x= & {} c\,\cos \beta \end{aligned}$$

5 $$\begin{aligned} c_y= & {} c\,(\cos \alpha - \cos \beta \, \cos \gamma )/\sin \gamma \end{aligned}$$

6 $$\begin{aligned} c_z=\, & {} \sqrt{c^2-c_x^2-c_y^2}. \end{aligned}$$

The monoclinic system is obtained by setting $\alpha =\gamma =\pi /2$,

7 $$\begin{aligned} \varvec{T_{\mathrm {m}}}= \left( \begin{array}{ccc} a &{}\quad 0 &{}\quad c\,\cos \beta \\ 0 &{}\quad b &{}\quad 0 \\ 0 &{}\quad 0 &{}\quad c\,\sin \beta \\ \end{array} \right) . \end{aligned}$$

The $\varvec{{\tilde{y}}}$-direction is perpendicular to the ($\varvec{{\tilde{x}}}$,$\varvec{{\tilde{z}}}$)-plane.

## Rotation transformation of the coordinates

The spherical angles $\theta$ and $\phi$ define the rotational transformation of vectors ${\varvec{r}}$ in the crystal system into vectors $\varvec{r'}$ in another Cartesian coordinate system. A rotation of the crystal is generally described by a rotation matrix *R*,

8 $$\begin{aligned} \varvec{r'} = {\varvec{R}} \, {\varvec{r}}, \end{aligned}$$

We consider the rotation around the $\varvec{{\tilde{z}}}$-axis by the angle $\phi$,

9 $$\begin{aligned} {\varvec{R}}_{z}(\phi )=\left( \begin{array}{ccc} \cos \phi &{}\quad -\sin \phi &{}\quad 0 \\ \sin \phi &{}\quad \cos \phi &{}\quad 0 \\ 0 &{}\quad 0 &{}\quad 1 \\ \end{array} \right) , \end{aligned}$$

and subsequently the rotation around the $\varvec{{\tilde{y}}}$-axis by the angle $\theta$,

10 $$\begin{aligned} {\varvec{R}}_{y}(\theta )=\left( \begin{array}{ccc} \cos \theta &{}\quad 0 &{}\quad \sin \theta \\ 0 &{}\quad 1 &{}\quad 0 \\ -\sin \theta &{}\quad 0 &{}\quad \cos \theta \\ \end{array} \right) . \end{aligned}$$

**Figure 1:**
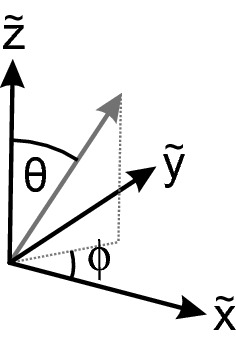
Schematic of Cartesian coordinate system $\varvec{{\tilde{x}}}$, $\varvec{{\tilde{y}}}$, $\varvec{{\tilde{z}}}$, with a crystal direction (grey arrow) and the angles $\theta$ and $\phi$. After the rotation according to Eq. (11), the grey arrow points along $\varvec{{\tilde{z}}}$.

An arbitrary direction can be generated with the combined rotation (Fig. 1)

11 $$\begin{aligned} {\varvec{R}}={\varvec{R}}_{y}(\theta ) \, {\varvec{R}}_{z}(\phi ). \end{aligned}$$

The angles have a useful range of $-\pi /2 \le \theta \le \pi /2$ and $0 \le \phi \le 2\pi$.

## Stress–strain relation in the crystal

The stress-strain relation in the crystal system reads

12 $$\begin{aligned} \varvec{\sigma }= {\varvec{C}} \, {\varvec{e}} \end{aligned}$$

with the stiffness components $C_{ij}$ for the 6-tuples of stress $\varvec{\sigma }$ and strain ${\varvec{e}}$ in the Voigt notation,

13 $$\begin{aligned} \varvec{\sigma }= & {} (\sigma _{11}, \sigma _{22}, \sigma _{33}, \sigma _{23}, \sigma _{13}, \sigma _{12})^{\mathrm {T}} \end{aligned}$$

14 $$\begin{aligned} {\varvec{e}}= & {} (\epsilon _{11}, \epsilon _{22}, \epsilon _{33}, 2\epsilon _{23}, 2\epsilon _{13}, 2\epsilon _{12})^{\mathrm {T}}. \end{aligned}$$

The (symmetrized) strain components are derived from the displacement ${\varvec{u}}$ via $\epsilon _{ij}=(\partial u_i/\partial x_j+\partial u_j/\partial x_i)/2$. The $6 \times 6$ matrix ${\varvec{C}}$ contains the elastic (stiffness) constants and is given with respect to the same $(\varvec{{\tilde{x}}}$, $\varvec{{\tilde{y}}}$, $\varvec{{\tilde{z}}})$ coordinate system as chosen in (7). The matrix ${\varvec{C}}$ is symmetric, i.e. $C_{ij}=C_{ji}$. For the triclinic system, all entries are non-zero, yielding 21 components; by special choice of coordinate system, the number can be reduced to 18 independent constants^1^. For the monoclinic system, 13 non-zero components remain; by special choice of coordinate system, the number can be reduced to 12 independent constants^1^. Special forms of ${\varvec{C}}$ are given for all crystals in^1,21^ and contain many zeros for suitable choices of coordinate system.

For a monoclinic material (mirror plane for $y=0$) (12) reads

15 $$\begin{aligned} {\varvec{C}}= \left( \begin{array}{cccccc} C_{11} &{}\quad C_{12} &{}\quad C_{13} &{}\quad 0 &{}\quad C_{15} &{}\quad 0 \\ C_{12} &{}\quad C_{22} &{}\quad C_{23} &{}\quad 0 &{}\quad C_{25} &{}\quad 0 \\ C_{13} &{}\quad C_{23} &{}\quad C_{33} &{}\quad 0 &{}\quad C_{35} &{}\quad 0 \\ 0 &{}\quad 0 &{}\quad 0 &{}\quad C_{44} &{}\quad 0 &{}\quad C_{46} \\ C_{15} &{}\quad C_{25} &{}\quad C_{35} &{}\quad 0 &{}\quad C_{55} &{}\quad 0 \\ 0 &{}\quad 0 &{}\quad 0 &{}\quad C_{46} &{}\quad 0 &{}\quad C_{66} \end{array} \right) \end{aligned}$$

The technicalities of the transformation of the matrix ${\varvec{C}}$ under rotation into ${\varvec{C}}'$ are discussed at length in^8,9^. We define $C_5=C_{15}+C_{25}+C_{35}$.

For monoclinic (and triclinic) materials, the special PA-R coordination system can be found for which

16 $$\begin{aligned} 0=\, & {} C'_4 = C'_{14}+C'_{24}+C'_{34} \end{aligned}$$

17 $$\begin{aligned} 0=\, & {} C'_5 = C'_{15}+C'_{25}+C'_{35} \end{aligned}$$

18 $$\begin{aligned} 0=\, & {} C'_6 = C'_{16}+C'_{26}+C'_{36} . \end{aligned}$$

Here, for isotropic dilation, i.e. $e_1=e_2=e_3$, without shear strains, i.e. $e_4=e_5=e_6=0$, the tangential forces vanish, i.e. $\sigma _4=\sigma _5=\sigma _6=0$ and it is evoked only by normal forces.

The reciprocal equation,

19 $$\begin{aligned} {\varvec{e}}= {\varvec{S}} \, \varvec{\sigma } \end{aligned}$$

contains the compliances $S_{ij}$ with ${\varvec{S}}={\varvec{C}}^{-1}$. For the rotated system, $\varvec{S'}=\varvec{C'}^{-1}$. The coordination system fulfilling equations (20)–(22) is the principal axes system of elastic deformation (PA-D).

20 $$\begin{aligned} 0= & {} S'_4 = S'_{14}+S'_{24}+S'_{34} \end{aligned}$$

21 $$\begin{aligned} 0= & {} S'_5 = S'_{15}+S'_{25}+S'_{35} \end{aligned}$$

22 $$\begin{aligned} 0= & {} S'_6 = S'_{16}+S'_{26}+S'_{36} . \end{aligned}$$

Here, for hydrostatic pressure, i.e. isotropic normal forces, $\sigma _1=\sigma _2=\sigma _3$ and $\sigma _4=\sigma _5=\sigma _6=0$, the shear strains vanish, i.e. $e_4=e_5=e_6=0$, meaning that a rectangular box with sides aligned to this coordinate system keeps its right angles

For any crystal except monoclinic or triclinic the two PA-D and PA-R coordinate systems coincide. Only for these two low symmetry crystal classes, they have different orientations. We note that a parameter (and criterion) for triclinicity has been given in^22^.

## Orientation of the principal axes system of elastic resistance (PA-R)

We look now for the angles of rotation of the PA-R system relative to the crystal system $(\varvec{{\tilde{x}}}, \varvec{{\tilde{y}}}, \varvec{{\tilde{z}}})$. In the monoclinic system for symmetry reasons, the angle $\phi$ must be zero and the rotation must lie around the $\varvec{{\tilde{y}}}$-axis. Also, if $\theta _0$ is a solution, $\theta _0+n\, \pi /2$, $n \in {\mathbb {Z}}_0$ must a solution as well. This will come out explicitly.

We assume that $C_5 \ne 0$, otherwise the solution is already $\theta =\phi =0$. In the rotated coordinate system, we find,

23 $$\begin{aligned} C'_4=\, & {} C_5 \, \cos \theta \, \sin \phi +p \, \sin \theta \, \cos \phi \, \sin \phi \end{aligned}$$

24 $$\begin{aligned} C'_5=\, & {} C_5 \, \cos 2 \theta \, \cos \phi + (q+p \, \cos 2 \phi )/4 \, \sin 2 \theta \end{aligned}$$

25 $$\begin{aligned} C'_6=\, & {} C_5 \, \sin \theta \, \sin \phi - p \cos \theta \, \cos \phi \, \sin \phi . \end{aligned}$$

with $q=-C_{11}-2 C_{12}+C_{13}-C_{22}+C_{23}+2 C_{33}$ and $p=-C_{11}-C_{13}+C_{22}+C_{23}$. From (23) and $C'_4=0$, we find $\phi =0$ and the same from (25) and $C'_6=0$. Then, (24) and $C'_5=0$ reads,

26 $$\begin{aligned} \cos 2 \theta _{\mathrm {C}} + \xi \, \cos \theta _{\mathrm {C}} \, \sin \theta _{\mathrm {C}}=0, \end{aligned}$$

with

27 $$\begin{aligned} \xi =\frac{-C_{11} - C_{12} + C_{23} + C_{33}}{C_{15}+C_{25}+C_{35}}. \end{aligned}$$

The index ’C’ indicates that this angle belongs to the system for which $C'_5=0$.

The solutions are

28 $$\begin{aligned} \theta _{\mathrm {C},\pm }=\arctan \left( \frac{\xi \pm \sqrt{4+\xi ^2}}{2} \right) + n \, \pi , \end{aligned}$$

with $n \in {\mathbb {Z}}_0$. We calculate the angular difference of $\theta _+$ and $\theta _-$; for $\xi =0$ one can see quickly that $\theta _+-\theta _-=\pi /4-(-\pi /4)=\pi /2$. The derivatives with respect to $\xi$ are the same, $\theta '_+=\theta '_-=1/(4+\xi ^2)$. Thus $(\theta _+-\theta _-)'=0$ and $\theta _+-\theta _-=\pi /2$ for all $\xi$.

Therefore, the solutions can be finally written as

29 $$\begin{aligned} \theta _{\mathrm {C}}=\arctan \left( \frac{\xi + \sqrt{4+\xi ^2}}{2} \right) + n \, \pi /2. \end{aligned}$$

We chose as solution the angle with the smallest absolute value, i.e. a value in the range $-\pi /4 \le \theta _{\mathrm {C}} \le \pi /4$. The principal axis system is then given by the directions $\theta _{\mathrm {C}}$ and $\theta _{\mathrm {C}}+\pi /2$ in the ($\varvec{{\tilde{x}}},\varvec{{\tilde{z}}}$)-plane and the $\varvec{{\tilde{y}}}$ direction.

## Orientation of the principal axes system of elastic deformation (PA-D)

Now we investigate the coordinate system for which $S'_5=0$. Again, we find $\phi =0$ from $S'_4=S'_6=0$. If $S_5=0$ already, $\theta _{\mathrm {S}}=0$ of course; the index ’S’ is used now for distinction. The calculation of the inverse of $\varvec{C'}$ delivers the condition

30 $$\begin{aligned} \cos 2 \theta _{\mathrm {S}} + \zeta \, \sin 2\theta _{\mathrm {S}}=0. \end{aligned}$$

with $\zeta$ expressed via the $C_{ij}$ by,

$$\begin{aligned} \zeta= & {} \left[ C_{15}^2 (-C_{22} + C_{23}) + C_{25}^2 C_{33} + C_{13} C_{25} C_{35}\right. \\&\quad -\, 2 C_{23} C_{25} C_{35} - C_{12} C_{35}^2 + C_{22} C_{35}^2 \\&\quad +\, C_{11} C_{25} (-C_{25} + C_{35}) + C_{15} (-C_{25} (C_{13} + C_{33}) \\&\quad +\, C_{12} (2 C_{25} - C_{35}) + C_{23} C_{35}) + C_{11} (C_{22} - C_{23}) C_{55} \\&\quad -\, (C_{12}- C_{23}) (C_{12} - C_{13} + C_{23}) C_{55} \\&\quad \left. +\, (C_{12} - C_{22}) C_{33} C_{55} \right] / \left[ C_{15} C_{23}^2 + C_{13}^2 C_{25} + C_{11} C_{23} C_{25} - C_{15} C_{22} C_{33} \right. \\&\quad -\, C_{11} C_{25} C_{33} + C_{12}^2 C_{35} + C_{11} (-C_{22} + C_{23}) C_{35} \\&\quad +\, C_{13} (C_{15} (C_{22} - C_{23}) - (C_{12} + C_{23}) C_{25} \\&\quad +\, (-C_{12} + C_{22}) C_{35}) \\&\quad \left. +\, C_{12} (-C_{15} C_{23} + C_{15} C_{33} + C_{25} C_{33} - C_{23} C_{35}) \right] . \end{aligned}$$

It should be mentioned that this formula does not depend on $C_{44}$, $C_{46}$ and $C_{66}$.

The solutions of (30) are given by,

31 $$\begin{aligned} \theta _{\mathrm {S}}=\frac{\arctan (-1/\zeta )}{2} + n\, \pi /2, \end{aligned}$$

$n \in {\mathbb {Z}}_0$. Again we chose $-\pi /4 \le \theta _{\mathrm {S}} \le \pi /4$. The principal axis system is then given by the directions $\theta _{\mathrm {S}}$ and $\theta _{\mathrm {S}}+\pi /2$ in the ($\varvec{{\tilde{x}}},\varvec{{\tilde{z}}}$)-plane and the $\varvec{{\tilde{y}}}$ direction.

## Numerical results for $\beta$-${\text {Ga}}_2 {\text {O}}_3$ and $\theta$-${\text {Al}}_2 {\text {O}}_3$

For monoclinic gallia and alumina various sets of elastic constants have been reported from density functional theory (DFT)^10–18,24^ , force-field simulation^23^ and for gallia in experiment^18,23^, as listed in Table 1.

**Table 1:** Elastic constants of monoclinic (C2/m) gallia and alumina (from^16^ the values for 0 K with zero-point vibrations) (in units of $10^{11}$ Pa) and angular positions of specific elastic properties as defined in the text (in degrees).

Material reference method	$\beta$-${\text {Ga}}_2 {\text {O}}_3$											$\theta$-${\text {Al}}_2 {\text {O}}_3$		
	^10^	^11^	^12^	^13^	^14^	^18^	^18^	^18^	^23^	^23^	^24^	^15^	^16^	^17^
	AM05	LDA	LDA	GGA	PBESOL	RUS/LDI	LDA	GGA	RUS	FFS	LDA	LDA	LDA	GGA
$C_{11}$	2.231	2.37	2.349	1.99	2.27	2.428	2.19	2.04	2.38	2.85	2.42	2.838	2.78	2.51
$C_{12}$	1.165	1.25	1.262	1.12	1.28	1.280	1.27	1.16	1.30	1.35	1.27	1.193	1.15	1.16
$C_{13}$	1.253	1.47	1.577	1.25	1.35	1.600	1.69	1.39	1.52	1.35	1.40	1.598	1.51	1.52
$C_{22}$	3.332	3.54	3.638	3.12	3.35	3.438	3.65	3.24	3.59	4.00	3.60	4.204	4.10	3.87
$C_{23}$	0.750	0.95	1.076	0.62	0.728	0.709	1.06	0.78	0.78	0.90	0.903	0.830	0.77	0.61
$C_{33}$	3.300	3.57	3.532	2.98	3.13	3.474	3.44	3.05	3.46	3.76	3.55	4.353	4.27	3.87
$C_{15}$	− 0.174	− 0.18	− 0.206	− 0.02	− 0.036	− 0.0162	− 0.014	− 0.013	− 0.04	− 0.13	− 0.177	− 0.307	− 0.29	− 0.01
$C_{25}$	0.122	0.11	0.083	0.01	0	0.0036	0.035	0.021	0.02	0.08	0.12	0.123	0.13	0.02
$C_{35}$	0.073	0.06	0.067	0.17	0.18	0.0097	0.18	0.17	0.19	− 0.35	0.077	0.167	0.16	0.22
$C_{46}$	0.174	0.19	0.214	0.03	0.064	0.0559	0.13	0.078	0.06	0.22	0.197	0.238	0.23	0.05
$C_{44}$	0.503	0.54	0.516	0.39	0.453	0.478	0.54	0.45	0.49	0.50	0.58	0.868	0.84	0.62
$C_{55}$	0.686	0.67	0.633	0.77	0.83	0.886	0.76	0.73	0.91	0.73	0.69	1.043	1.04	1.19
$C_{66}$	0.942	0.95	0.907	0.95	0.99	1.040	0.99	0.93	1.07	0.93	0.97	1.245	1.24	1.28
$\theta _{\mathrm {C}}$	− 1.84	0.64	3.21	− 16.6	− 21.5	0.35	− 10.6	− 14.7	− 15.6	30.1	− 1.05	0.85	0	− 14.8
$\theta _{\mathrm {S}}$	5.60	7.14	10.0	− 8.85	− 12.8	0.69	− 3.85	− 7.55	− 8.03	33.3	5.77	7.73	6.81	− 7.85
$\theta _{\mathrm {S}}$-$\theta _{\mathrm {C}}$	7.44	6.50	6.79	7.75	8.7	0.34	6.75	7.15	7.57	3.2	6.82	6.88	6.81	6.95
$\theta _{\mathrm {Y,min}}$	19.2	19.8	19.7	5.3	6.3	1.1	5.5	5.6	6.0	42.9	20.5	17.1	17.0	3.4
$\theta _{\mathrm {Y,max}}$	79.3	79.8	77.6	65.7	65.5	61.8	63.9	65.2	64.7	103.8	80.0	74.5	75.0	62.1

AM05: generalized gradient functional^25^, GGA: generalized gradient approximation, LDA: local density approximation, PBESOL: gradient functional^26^, RUS/LDI: resonant ultrasound spectroscopy, LDI: laser-Doppler interferometry, FFS: force-field simulation^27^.

**Figure 2:**
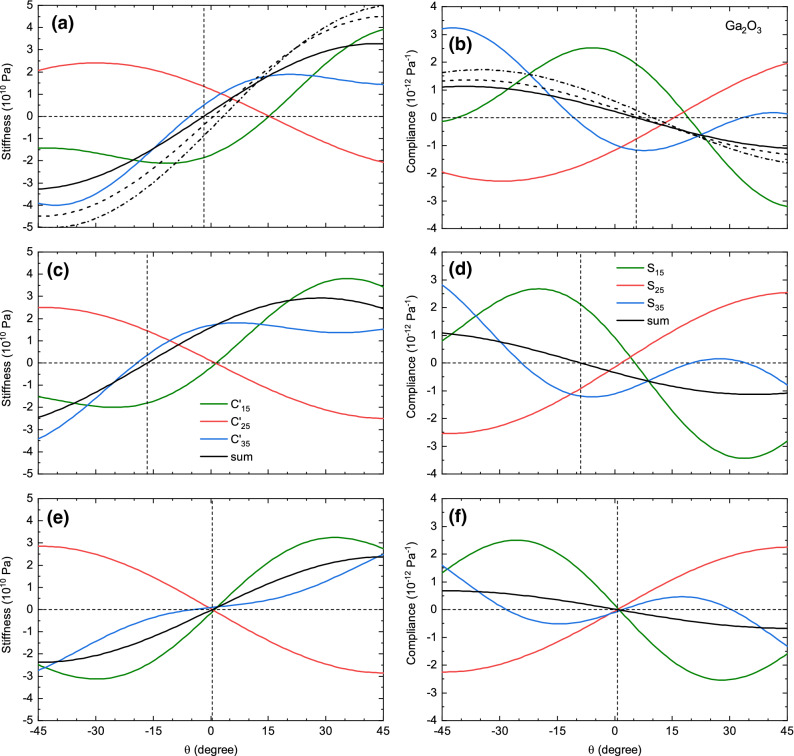
Comparison of the angular dependence of (**a**, **c**, **e**) $C_{15}$ (green), $C_{25}$ (red), $C_{35}$ (blue) and their sum (black) and of (**b**, **d**, **f**) $S_{15}$ (green), $S_{25}$ (red), $S_{35}$ (blue) and their sum (black) for various data sets of elastic constants of $\beta$-${\text {Ga}}_2 {\text {O}}_3$ from (**a**, **b**)^10^, (**c**, **d**)^13^, and (**e**, **f**)^18^. Also, the sums according to^11,12^ are depicted as black dashed (dash-dotted) lines in (**a**, **b**). The vertical dashed lines indicate the zeros of the black solid line sums.

For these sets we have calculated the angles $\theta _{\mathrm {C}}$ of the PA-R and $\theta _{\mathrm {S}}$ for the PA-D system as depicted in Fig. 2. Foremost, all calculations arrive at $\theta _{\mathrm {S}} \ne \theta _{\mathrm {C}}$, as expected for monoclinic material. The difference $\theta _{\mathrm {S}}-\theta _{\mathrm {C}}$ is within about one degree approximately 7$^{\circ }$ for all calculations (except FFS), showing that the effect is present but not drastic. For $\beta$-${\text {Ga}}_2 {\text {O}}_3$, several independent DFT calculations agree within a few degrees^10–12^ that $\theta _{\mathrm {C}}$ is close to zero. The absolute angles derived from^13^ (^14^) deviate a lot by about $17^{\circ }$ ($22^{\circ }$) from these publications, but several theories yield values around 15 degrees.

For ${\text {Al}}_2 {\text {O}}_3$, two calculations both from the same group^15,16^, deviate from^17^ also significantly by about $14{-}16^{\circ }$.

The experimental data for $\beta$-${\text {Ga}}_2 {\text {O}}_3$ from^18^ yield $\theta _{\mathrm {C}}$ close to zero, but also $\theta _{\mathrm {S}}$ is found close to zero; thus orientations of the PA-D and PA-R systems are almost identical, increasing the elastic symmetry. Approximately (and within the experimental error), for $C'_5=0$, also $C'_{25}=0$, i.e. $C'_{15}=-C'_{35}$. This is in contrast to all available DFT calculations where for $C'_5=0$, clearly none of the $C'_{i5}$ components ($i=1,2,3$) is zero. The experimental data for $\beta$-${\text {Ga}}_2 {\text {O}}_3$ from^23^ yield an angular difference between the PA-D and PA-R systems of about $7.6^{\circ }$, in agreement with most theories; the absolute angles are closest to the results of^13^.

## Young’s module

**Figure 3:**
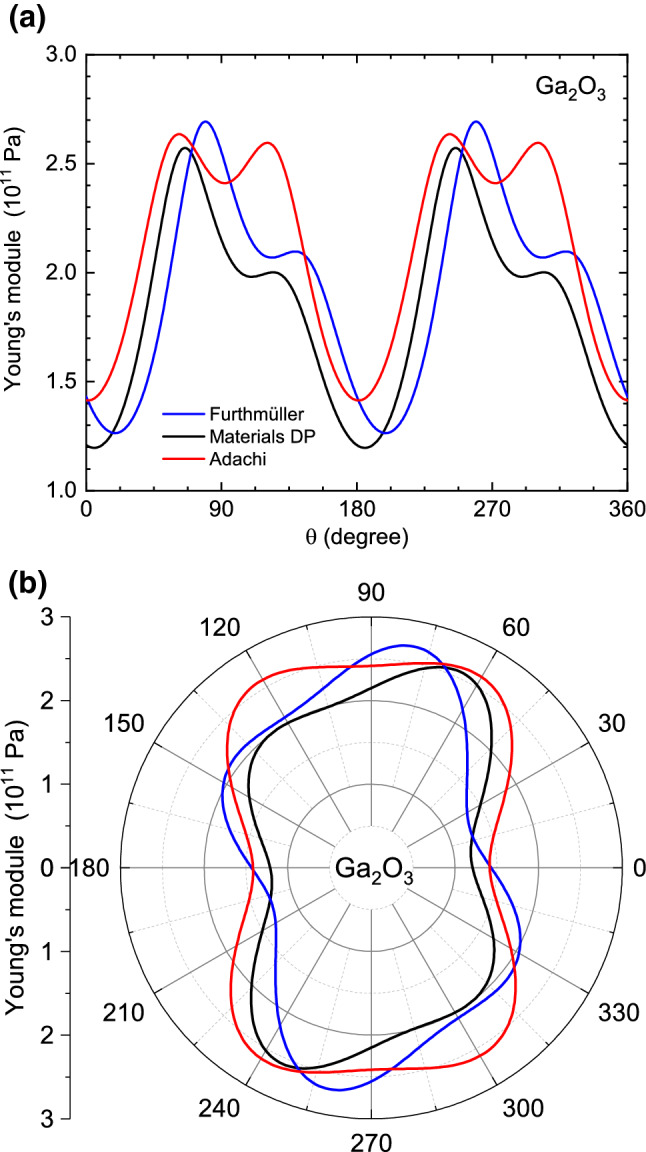
Young’s module of $\beta$-${\text {Ga}}_2 {\text {O}}_3$ in the (010)-plane ($\phi$=0) as a function of the rotation angle $\theta$ for three selected data sets from^10^ (blue),^13^ (black) and^18^ (experimental elastic constants, red).

The monoclinic angle $\beta \ne \pi /2$ also leads to a characteristic distortion of the angular dependence of the Young’s module $Y'=1/S'_{11}$ in the ($\varvec{{\tilde{x}}}$,$\varvec{{\tilde{z}}}$)-plane, i.e. the (010) crystallographic plane, away from mirror symmetries that are present for an orthorhombic system. We note that a three-dimensional view of the data from^13^ can be found in Ref.^28^. The remaining symmetry is that $Y'(\theta )=Y'(\theta +\pi )$. The angular dependence in the ($\varvec{{\tilde{x}}},\varvec{{\tilde{z}}}$)-plane is visualized in Fig. 3 for three data sets with linear angular scale and as polar plot. The angular positions $\theta _{\mathrm {Y,max}}$ and $\theta _{\mathrm {Y,min}}$ of the maximum and minimum values of the Young’s module, respectively, in the ($\varvec{{\tilde{x}}},\varvec{{\tilde{z}}}$)-plane, are listed in Table 1. There seems to be significant disagreement between different theories. The two experimental data sets yield rather similar values which agree more or less with theories in^13,14^. Notably, the theory of^23^ is the only one yielding $\theta _{\mathrm {Y,max}}>\pi /2$.

## Summary

We have presented analytical formulas for the orientations of the two symmetry-adapted Cartesian coordinate systems of monoclinic crystals, namely the compression and resistance ellipsoids. Various theoretical and experimental data sets for monoclinic gallia and alumina have been analyzed and significant differences between theories and theories and experiment have been found, making further investigations necessary to correctly capture the anisotropic elastic properties of these technologically important materials.

The data that support the findings of this study are available from the corresponding author upon reasonable request.
